# Emerging Therapies in Inflammatory Bowel Disease: A Comprehensive Review

**DOI:** 10.3390/jcm14176119

**Published:** 2025-08-29

**Authors:** John K. Appiah, Umar Hayat, Nikita Garg, Richeal Asante, Evans Donneyong, Muhammad U. Haider, Pranav Patel, Zubair Khan, Ali A. Siddiqui

**Affiliations:** 1 Department of Internal Medicine, Geisinger Wyoming Valley Medical Center, Wilkes-Barre, PA 18711, USA; jkappiah@geisinger.edu (J.K.A.); ngarg@geisinger.edu (N.G.); rasante@geisinger.edu (R.A.); usmanhaidermd@gmail.com (M.U.H.); 2 Department of Internal Medicine, Tamale Teaching Hospital, Tamale NT2701, Ghana; edonneyong@yahoo.com; 3 Department of Internal Medicine, Division of Gastroenterology, Geisinger Health System, Danville, PA 18711, USA; ppatel7@geisinger.edu; 4 Department of Gastroenterology, Advent Health, Orlando, FL 32803, USA; zubairkhan254@gmail.com; 5 Department of Gastroenterology, Maimonides Medical Center, New York, NY 11219, USA; asiddiqu2004@gmail.com

**Keywords:** Inflammatory bowel disease (IBD), Crohn’s disease (CD), ulcerative colitis (UC), Emerging therapies, Biological therapies

## Abstract

Inflammatory bowel disease (IBD), comprising Crohn’s disease (CD) and ulcerative colitis (UC), represents a significant challenge in gastroenterology due to its chronic nature, unpredictable course, and impact on patients’ quality of life. The therapeutic landscape for IBD has evolved significantly with the advent of biologic agents targeting specific immune pathways. However, limitations, including partial efficacy, side effects, and development of resistance, highlight the ongoing need for innovative treatment approaches. This review explores emerging therapies in IBD, including novel biologics, small molecules, microbiome-based therapies, and gene and stem cell therapies. The article summarizes their mechanisms of action, clinical efficacy, safety profiles, and potential future directions in IBD management. Methods: This comprehensive narrative review synthesizes current knowledge and emerging developments in inflammatory bowel disease (IBD) therapeutics. Literature was identified through targeted selection of high-quality sources, including pivotal randomized controlled trials, systematic reviews and meta-analyses, regulatory approval documents, and clinical practice guidelines from major gastroenterology societies. Emphasis was placed on recent publications (2020–2024) to capture the rapidly evolving therapeutic landscape, with particular attention to FDA/EMA-approved therapies and promising late-stage investigational agents. Sources were prioritized based on clinical relevance, study quality, and regulatory status. This narrative approach was selected to provide comprehensive coverage of diverse therapeutic modalities spanning conventional treatments to cutting-edge techniques, including biologics, small molecules, microbiome-based therapies, gene therapy, and stem cell treatments. The review acknowledges the inherent limitations of non-systematic literature selection while prioritizing clinical utility and educational value for healthcare providers managing IBD patients in contemporary practice.

## 1. Introduction

Inflammatory bowel disease (IBD), which includes Crohn’s disease (CD) and ulcerative colitis (UC), is a complex, chronic condition characterized by inflammation of the gastrointestinal tract [1]. The prevalence of IBD has increased globally, particularly in developed countries, and it affects over 6.8 million individuals worldwide, with healthcare costs exceeding $31 billion annually in the United States alone [2,3]. Recent epidemiological studies from 2022–2024 have revealed concerning trends, with pediatric-onset disease increasing by 15% over the past decade and early-onset IBD now representing 60% of all new diagnoses [4,5].

Although etiology remains multifactorial, involving genetic, environmental, and immunological factors, recent breakthroughs utilizing single-cell RNA sequencing have provided unprecedented insights into disease heterogeneity [6,7,8]. The emergence of post-infectious IBD following COVID-19 and pandemic-related lifestyle changes has added new dimensions to our understanding of environmental triggers [9,10].

The treatment of IBD has traditionally involved corticosteroids, immunosuppressants, and 5-aminosalicylates [11]. Biologic therapies have significantly changed the landscape of treatment for moderate-to-severe disease, particularly in patients who are refractory to conventional therapies [1,11]. However, these treatments are associated with many limitations, including high costs, side effects, and loss of efficacy over time, with primary non-response rates remaining at 30–40% and secondary loss of response affecting 13% of patients annually [1,11,12]. The concept of “treat-to-target” has evolved beyond clinical remission to encompass transmural healing, histologic normalization, and prevention of long-term disability [13,14].

Consequently, there is an urgent need for newer therapies with better safety profiles and sustained effectiveness [11]. Advances in artificial intelligence and machine learning applications in IBD management, including predictive modeling for treatment response, have opened new avenues for personalized therapy selection [15,16].

In this review, we explore the current state of IBD treatment; discuss novel biologic therapies, small-molecule treatments, and microbiome-based approaches; and examine the potential of gene and stem cell therapies for future management.

## 2. Pathophysiology

The pathogenesis of inflammatory bowel disease (IBD) is multifactorial, involving complex interactions among genetic susceptibility, environmental influences, immune dysregulation, and alterations in the gut microbiota [17]. While the exact etiology remains incompletely understood, advances in genomics and immunology have significantly improved our understanding of disease mechanisms [18].

Genetic predisposition plays a substantial role in IBD, particularly in Crohn’s disease (CD). Genome-wide association studies (GWAS) have identified over 240 risk loci associated with IBD, many implicated in pathways regulating innate and adaptive immunity, autophagy, microbial recognition, and epithelial barrier integrity [18,19]. Recent genome-wide studies published in 2023 have expanded our understanding to include novel pathways involving trained immunity and metabolomic signatures [20,21]. Notable genes include NOD2, IL23R, ATG16L1, and STAT3, contributing to host–microbiota interactions and immune homeostasis [19].

Environmental factors such as a Western diet, smoking, antibiotic exposure, and early-life infections are essential contributors. These factors can influence gut permeability, immune activation, and microbiota composition, potentially triggering disease onset in genetically predisposed individuals [22]. Recent studies have highlighted the role of ultra-processed foods and artificial sweeteners in IBD development [23,24].

**Figure 1:**
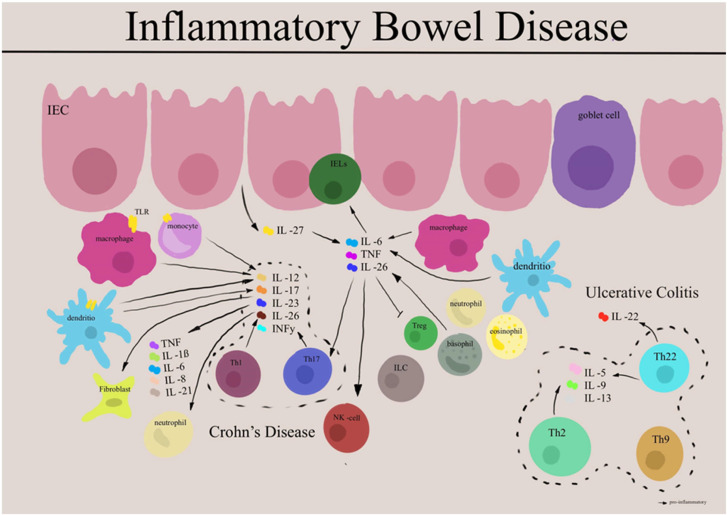
Cytokine Networks in Inflammatory Bowel Disease Pathogenesis. Schematic representation of key immune pathways in IBD. Intestinal epithelial cells (IEC) interact with immune cells, including dendritic cells, macrophages, and T cells. Crohn’s disease (**left**) shows Th17-predominant responses with IL-17, IL-22, and TNF-α production, while ulcerative colitis (**right**) demonstrates Th2-skewed immunity with IL-5 and IL-13 expression. Neutrophil infiltration and cytokine networks drive chronic inflammation in both conditions. Therapeutic targets include TNF-α, IL-12/IL-23, and JAK-STAT pathways. Adapted from [18].

Immune dysregulation is central to IBD pathogenesis. Immune dysregulation is central to IBD pathogenesis, with distinct cytokine profiles characterizing different disease subtypes. Crohn’s disease demonstrates Th1- and Th17-predominant responses, while ulcerative colitis shows atypical Th2-type inflammation [25]. Recent research has identified crucial roles for innate lymphoid cells and tissue-resident memory T cells in maintaining chronic inflammation, with these cell populations providing both rapid inflammatory responses and immunological memory that perpetuates disease chronicity [26,27] Figure 1.

The intestinal microbiome plays a pivotal role in maintaining gut homeostasis. In IBD, dysbiosis characterized by reduced microbial diversity, depletion of protective commensals such as Faecalibacterium prausnitzii, and overgrowth of pathobionts disrupts mucosal immune tolerance and contributes to chronic inflammation. This is often accompanied by epithelial barrier breakdown, allowing microbial products to translocate and further activate immune responses [28]. Recent metagenomic studies have identified specific bacterial strains and metabolites responsible for inflammatory processes [29,30].

Understanding these interrelated pathways provides a framework for targeted therapies, many of which aim to restore immune balance, reinforce barrier function, or correct microbial dysbiosis [18].

## 3. Current Treatment Landscape

### 3.1. Conventional Therapies

Managing inflammatory bowel disease (IBD) has traditionally relied on a combination of anti-inflammatory agents, corticosteroids, and immunosuppressive therapies. These agents reduce intestinal inflammation, induce clinical remission, and prevent disease complications. However, their use is often limited by variable efficacy, safety concerns, and long-term tolerability issues [31].

**5-Aminosalicylates (5-ASA)** are among the first-line agents for mild-to-moderate ulcerative colitis (UC). Drugs such as mesalamine, sulfasalazine, and olsalazine act topically on the colonic mucosa to reduce inflammation, primarily by inhibiting cyclooxygenase and lipoxygenase pathways [32]. These agents are less effective in Crohn’s disease (CD), where inflammation frequently extends beyond the reach of topical 5-ASA delivery. Although generally well tolerated, their use in CD is limited due to minimal clinical benefit in most patients [32].

**Corticosteroids** such as prednisone and budesonide are potent anti-inflammatory agents that induce remission during acute disease flares. They modulate multiple inflammatory pathways, including cytokine production and T-cell activation. Despite their effectiveness in controlling symptoms, long-term use is discouraged because of significant side effects, including osteoporosis, glucose intolerance, hypertension, adrenal suppression, and increased risk of infections [33]. Recent guidelines emphasize steroid-free remission as a key treatment goal [34].

**Immunosuppressive agents**, including thiopurines (azathioprine and 6-mercaptopurine) and methotrexate, are employed primarily as steroid-sparing agents and for maintenance of remission in steroid-dependent or refractory IBD. Their mechanisms involve suppression of purine synthesis and inhibition of lymphocyte proliferation. However, these agents have a delayed onset of action, often requiring several months, and are associated with adverse effects such as bone marrow suppression, hepatotoxicity, pancreatitis, and increased risk of lymphoproliferative disorders [35]. Pharmacogenomic testing for thiopurine metabolism is now recommended to optimize dosing and minimize toxicity [36].

While these conventional therapies continue to play a role, particularly in milder disease or as adjunctive agents, the limitations in durability of response and safety profiles have driven the development of biologics and small-molecule therapies that offer more targeted immunomodulation [31].

### 3.2. Biologic Therapies

Biologic therapies have significantly advanced the treatment of moderate to severe inflammatory bowel disease (IBD), offering targeted inhibition of specific inflammatory pathways through sophisticated mechanisms that represent a paradigm shift from conventional immunosuppression. These agents include monoclonal antibodies directed against tumor necrosis factor-alpha (TNF-α), integrins, and interleukins involved in the inflammatory cascade, each providing distinct therapeutic approaches based on their unique mechanisms of action and patient-specific considerations [37].

Anti-TNF agents, including infliximab, adalimumab, and certolizumab pegol, represent the most extensively studied biologic class in IBD, targeting TNF-α, a key cytokine in the pathogenesis of both Crohn’s disease (CD) and ulcerative colitis (UC). These agents effectively induce and maintain remission, heal mucosa, and reduce hospitalization and surgery rates through their mechanism of neutralizing both soluble and membrane-bound TNF-α, leading to reduced inflammatory cell activation, decreased cytokine cascade amplification, and induction of regulatory immune responses [38] Table 1. Clinical experience demonstrates that response patterns vary significantly based on patient characteristics, with factors such as age, disease phenotype, inflammatory markers, and concomitant medications influencing treatment outcomes. However, up to one-third of patients do not respond to induction therapy, termed primary non-response, and many lose response over time due to anti-drug antibody formation or pharmacokinetic variability, known as secondary loss of response [38]. The development of immunogenicity represents a critical limitation occurring in a substantial proportion of patients, depending on the specific agent and concurrent immunosuppression, necessitating proactive monitoring and optimization strategies to maintain long-term efficacy. Recent studies have emphasized the importance of therapeutic drug monitoring to optimize outcomes, with evidence suggesting that maintaining adequate drug exposure correlates with improved clinical and endoscopic outcomes, leading to personalized dosing approaches that may achieve superior remission rates in appropriately selected patients [39,40]. Optimal therapeutic outcomes appear to correlate with proactive drug level monitoring compared to reactive approaches, with clinical studies demonstrating that such strategies may improve long-term treatment durability [40].

Anti-integrin therapy includes vedolizumab, a monoclonal antibody that selectively blocks α4β7 integrin, thereby preventing lymphocyte trafficking to the gut mucosa through a mechanism that specifically targets gut-homing immune cells while preserving systemic immune function. This gut-selective mechanism results in a favorable safety profile, particularly due to the reduced risk of systemic immunosuppression, making it an attractive option for patients with increased infection risk or those requiring long-term immunomodulation [41]. Clinical experience suggests that this gut-selective approach may provide favorable safety profiles while maintaining therapeutic efficacy, though onset of action may differ from systemic immunosuppressive approaches, with some patients experiencing gradual improvement over several months rather than rapid response [41]. Vedolizumab’s unique mechanism offers potential advantages in specific patient populations, particularly elderly patients or those with comorbidities that increase susceptibility to systemic immunosuppression complications. A non-selective anti-integrin, natalizumab, is used less frequently in IBD because of its association with progressive multifocal leukoencephalopathy (PML), highlighting the importance of selectivity in integrin targeting [41]. Real-world studies have confirmed the long-term safety and efficacy of vedolizumab, with accumulating evidence supporting sustained benefit and confirming the favorable safety profile observed in clinical trials [42]. Patient selection considerations increasingly recognize that vedolizumab may be particularly suitable for certain clinical scenarios, including patients with systemic immunosuppression concerns or those requiring combination with other immunomodulatory therapies [41].

Anti-IL-12/23 therapy is represented by ustekinumab, which targets the p40 subunit shared by IL-12 and IL-23, modulating Th1 and Th17 pathways implicated in both CD and UC through a mechanism that addresses key cytokine networks central to IBD pathogenesis. Clinical trials have demonstrated efficacy in patients with prior biologic failure or intolerance, suggesting that this mechanism may provide therapeutic benefit even in treatment-experienced populations where other approaches have been unsuccessful [43]. The dual targeting of IL-12 and IL-23 pathways offers a broader immunomodulatory approach compared to more selective interventions, potentially addressing multiple inflammatory cascades simultaneously. Recent post-marketing surveillance data support its favorable long-term safety profile, with accumulating real-world evidence confirming the safety observations from controlled clinical trials and suggesting sustained benefit with prolonged use [44]. Clinical experience has identified patient populations that may be particularly suitable for ustekinumab therapy, including those with previous anti-TNF failure, patients requiring favorable safety profiles, and those with specific disease phenotypes that may respond preferentially to IL-12/23 pathway modulation.

The advent of biosimilars represents a significant advancement in biologic therapy accessibility, as these products are highly similar to approved originator biologics and have provided cost-effective alternatives without compromising efficacy or safety. Biosimilars of infliximab and adalimumab are increasingly adopted in both treatment-naïve patients and those transitioning from originator products, with regulatory approval based on comprehensive analytical, preclinical, and clinical comparability studies [45]. Recent studies have demonstrated successful switching between originator and biosimilar products, with clinical experience supporting the concept that appropriately approved biosimilars provide equivalent therapeutic outcomes while potentially improving access through reduced costs [46]. The availability of biosimilars has transformed the economic landscape of biologic therapy, potentially enabling broader patient access to these effective treatments while maintaining therapeutic standards.

Despite these significant advances, important challenges persist in biologic therapy. These include substantial variability in patient response patterns, with individual patients demonstrating different response kinetics and durability even within the same therapeutic class. The risk of immunogenicity remains a concern across all biologic agents, potentially leading to loss of efficacy and requiring ongoing monitoring and management strategies. High treatment costs continue to represent barriers to access and healthcare sustainability, though biosimilar availability is beginning to address this challenge. The requirement for parenteral administration presents logistical challenges and may affect patient preferences and adherence compared to oral alternatives. Such limitations highlight the ongoing need for next-generation therapies that offer greater precision through improved patient selection strategies, enhanced tolerability through reduced immunogenicity and adverse effects, and more convenient delivery options including oral formulations and extended dosing intervals [37]. Future developments in biologic therapy are likely to focus on personalized treatment approaches, combination strategies, and novel delivery mechanisms that can overcome current limitations while building upon the substantial therapeutic advances already achieved.

### 3.3. Small-Molecule Therapies

Small molecules offer the advantage of oral administration, unlike biologics, which are typically delivered via injection or infusion. These therapies target key intracellular pathways involved in the inflammatory process and provide an alternative to biologics, particularly in patients who have not responded to or are ineligible for biologic treatment Table 2 [47].

**Janus kinase (JAK) inhibitors** such as tofacitinib have demonstrated efficacy in ulcerative colitis (UC), particularly in patients who have failed conventional therapies or biologics [48]. By inhibiting the JAK-STAT signaling pathway, tofacitinib blocks the transmission of signals from pro-inflammatory cytokines, including interleukins (IL-2, IL-4, IL-6) and interferons (IFN-γ) [49]. It has shown significant effectiveness in inducing and maintaining remission in UC. Recent meta-analyses have supported the efficacy and safety of JAK inhibitors in clinical and endoscopic remission [50,51]. However, its use is associated with concerns about long-term safety, including increased risks of infections, thrombosis, and malignancy. These risks have led to close monitoring during therapy, and the drug is typically reserved for patients with prior treatment failure [49]. Newer selective JAK inhibitors like filgotinib and upadacitinib have shown promise with potentially improved safety profiles [52,53].

**Sphingosine 1-phosphate receptor (S1PR) modulators** such as ozanimod and etrasimod selectively prevent the egress of lymphocytes from lymph nodes, thereby reducing the inflammatory response in the gut. Ozanimod has demonstrated promising efficacy in UC, showing favorable safety and effectiveness in moderate to severe disease [54]. Like JAK inhibitors, S1PR modulators are administered orally, which may improve patient adherence compared to biologics that require parenteral delivery. Early evidence also suggests efficacy in patients who have previously failed anti-TNF or other biologic therapies [54]. Etrasimod recently received FDA approval for UC treatment based on positive Phase III results [55].

These small molecules, with their oral administration and targeted mechanisms of action, are becoming increasingly important in the therapeutic arsenal for IBD. However, their long-term safety profiles remain an active area of investigation, particularly concerning immunosuppression and malignancy risk [47].

### 3.4. Precision Medicine and Treatment Selection

Given the apparent therapeutic ceiling affecting current drug classes and the recognition of significant inter-patient variability in treatment response, optimal IBD management increasingly requires precision medicine approaches that integrate patient-specific factors with mechanistic considerations. Current clinical implementation of pharmacogenomic biomarkers includes well-established TPMT and NUDT15 genotyping for thiopurine dosing, which represents a strongly recommended approach that can prevent severe myelotoxicity through appropriate dose adjustments [36]. Emerging applications in pharmacogenomics research suggest potential utility of genetic variants in predicting response to various therapeutic classes, though clinical implementation awaits further validation [36]. Immunologic profiling approaches are being investigated for mechanism selection, where cytokine signatures may help guide therapy choice, with preliminary evidence suggesting that different inflammatory patterns might favor specific therapeutic approaches [25,26].

Microbiome-based selection represents an evolving area of research, with studies investigating whether baseline microbial signatures can predict treatment response. Research has examined whether specific bacterial populations or diversity patterns correlate with therapeutic outcomes, though clinical application requires further validation [28,29,30]. First-line biologic selection increasingly considers patient-specific factors beyond disease severity. Clinical experience suggests that patient age, comorbidity burden, infection risk, and disease phenotype may influence optimal therapy choice [31,37]. Current guidelines and clinical experience support individualized approaches where younger patients with inflammatory disease patterns may respond differently than older patients with complications or comorbidities [31,34].

The choice between small molecules and biologics depends on multiple patient factors, including administration preferences, previous treatment responses, and individual risk profiles. JAK inhibitors offer oral administration and potentially more rapid onset compared to biologics, while biologics may provide different safety profiles and mechanisms of action [47,48]. Previous treatment experience significantly influences selection strategies, with different approaches recommended for treatment-naive patients versus those with prior biologic exposure [31,37]. Evidence suggests that patients with primary non-response to one mechanism may benefit from alternative pathways. In contrast, those with secondary loss of response might benefit from optimization strategies before mechanism switching [38,41].

Therapeutic drug monitoring has emerged as an important tool for optimizing biologic therapy, with studies demonstrating that drug level optimization can improve clinical outcomes [39,40]. Research supports the concept that maintaining adequate drug exposure correlates with better therapeutic response, leading to increased utilization of proactive monitoring approaches [39]. Clinical guidelines increasingly emphasize the importance of drug level assessment in managing patients with suboptimal response or loss of efficacy [40]. Different biologics may have varying requirements for monitoring frequency and target levels, with accumulating evidence supporting individualized dosing based on drug level assessments rather than standard population-based approaches [39,40].

Special population considerations require tailored treatment approaches. Clinical experience and safety data suggest that elderly patients may benefit from therapies with favorable safety profiles and reduced systemic immunosuppression [55]. Patients with comorbidities, including infection history, malignancy concerns, or cardiovascular disease, require careful risk-benefit assessment when selecting among available therapeutic options. Treatment-experienced patients represent a particularly challenging population where sequential therapy decisions must consider the mechanism of previous failure, duration of response, and individual patient factors that may influence subsequent treatment choices [31,37].

## 4. Emerging Therapies in IBD

### 4.1. Next-Generation Biologics

Several new biologics are under investigation in inflammatory bowel disease (IBD), focusing on cytokine-targeting therapies and more precise immunomodulation. These next-generation biologics aim to provide greater specificity in targeting the underlying disease mechanisms, potentially offering more effective treatments with fewer side effects than traditional therapies [56].

**Figure 2:**
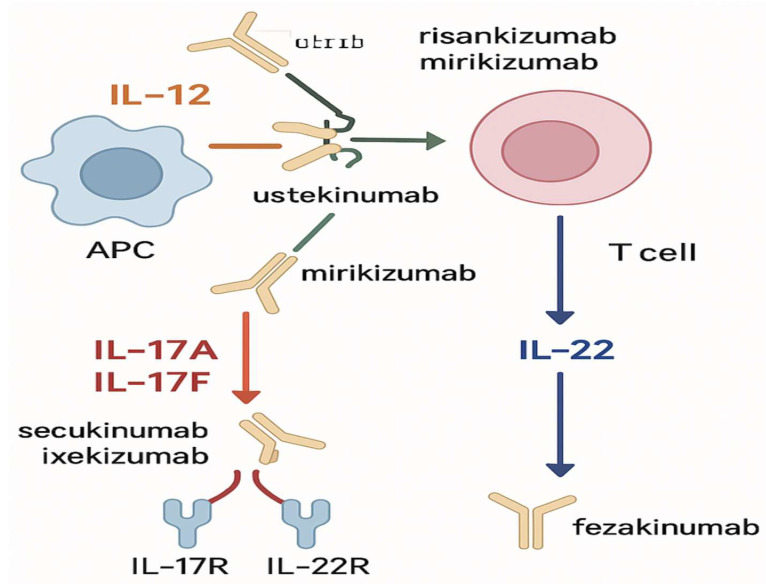
Targets of Emerging Biologic Therapies in IBD.

**Anti-IL-17 therapies** focus on interleukin-17 (IL-17), a pro-inflammatory cytokine involved in the pathogenesis of IBD, particularly Crohn’s disease (CD). Secukinumab and brodalumab are monoclonal antibodies specifically targeting IL-17A and have been evaluated in clinical trials for their potential in treating CD. Early results demonstrated reductions in disease activity and improvement in mucosal healing, but outcomes have been mixed, with some studies showing disease exacerbation [57,58] Figure 2. The use of IL-17 inhibitors represents targeting different immune pathways beyond those traditionally addressed by TNF-α or IL-12/23 blockade. However, further studies are needed to confirm long-term efficacy and safety, especially regarding the risk of opportunistic infections and exacerbation of other autoimmune diseases [57,58].

**Anti-IL-23 therapies** offer another innovative approach. IL-23 is critical in differentiating and activating T-helper 17 (Th17) cells, which are central to IBD pathogenesis. Guselkumab and mirikizumab are monoclonal antibodies targeting the p19 subunit of IL-23, providing more selective inhibition compared to ustekinumab. Guselkumab has shown promise in early-phase studies for both ulcerative colitis (UC) and CD [59]. Mirikizumab has demonstrated significant efficacy in Phase III trials for UC, with FDA approval received in 2023 [60,61]. By selectively inhibiting IL-23, these agents reduce the inflammatory activity of Th17 cells, providing a novel mechanism of action distinct from anti-TNF agents [59]. Early clinical trials have demonstrated meaningful reductions in disease activity and improved quality of life for patients with moderate to severe IBD [59].

**Dual targeting therapies** represent a new direction in IBD treatment. Combining biologics that block different immune pathways, such as pairing anti-TNF agents with anti-IL-23 antibodies, is being investigated to enhance treatment efficacy, particularly in refractory or severe cases [62]. This strategy is based on the rationale that targeting both TNF-α, a key pro-inflammatory cytokine, and IL-23, a Th17 cell activator, may result in more comprehensive immune modulation and improved inflammation control. Bispecific antibodies targeting multiple pathways simultaneously are also in development [63]. Preliminary data suggest this dual therapy may lead to higher clinical remission and mucosal healing rates. Nevertheless, concerns about cumulative immunosuppression and increased infection risk require careful long-term evaluation [62].

### 4.2. Fecal Microbiota Transplantation (FMT)

Fecal microbiota transplantation (FMT) has gained increasing attention as a potential therapeutic option for inflammatory bowel disease (IBD), particularly UC, representing a paradigm shift toward microbiome-targeted interventions. FMT involves transferring stool from a healthy donor into the gastrointestinal tract of a patient with IBD to restore a balanced and diverse intestinal microbiota that may be disrupted in these patients. The underlying rationale is based on the growing evidence that dysbiosis, an imbalance in the gut microbial community, plays a significant role in the pathogenesis and progression of IBD [64,65].

Recent systematic reviews and meta-analyses provide more measured evidence regarding FMT efficacy in UC. The most comprehensive meta-analysis by El Hage Chehade et al. [66] analyzing double-blind randomized controlled trials, reported a pooled clinical remission rate of 28% (95% CI: 16–43%) for FMT versus 9% (95% CI: 4–19%) for placebo, yielding a risk ratio of 1.70 (95% CI: 1.12–2.56, *p* = 0.01) [67]. While these results suggest potential benefit, the absolute difference in remission rates remains modest at approximately 20%.

The evidence for FMT in Crohn’s disease (CD) remains limited and inconclusive. A systematic review found insufficient high-quality data to support routine clinical use in CD. The therapeutic effect appears to be influenced by various factors, including the route of administration, such as colonoscopy, enema, or oral capsules; frequency of FMT delivery; donor selection; and the baseline microbial composition of the recipient [68,69]. Response rates vary significantly across studies, ranging from 13–44%, highlighting the need for standardized protocols and patient selection criteria.

Another meta-analysis by Ianiro et al. [70] demonstrated a pooled odds ratio for clinical remission of 1.87 (95% CI: 1.18–2.95) when comparing FMT to placebo. The heterogeneity in disease presentation and involvement of deeper layers of the intestinal wall in CD may contribute to the inconsistent outcomes observed across studies. Small pilot studies have documented symptom improvement in some CD patients following FMT, but remission rates have not exceeded those seen with conventional therapies [70,71].

Recent advances include the development of standardized frozen FMT preparations and encapsulated formulations, which may improve accessibility and standardization [71,72]. However, optimal protocols remain undefined, including donor selection criteria and screening protocols, standardized preparation and processing methods, optimal delivery routes and treatment frequency, patient selection algorithms and biomarker identification, and long-term safety monitoring protocols. Despite promising findings in selected UC patients, several important limitations persist. The modest effect sizes yield a number needed to treat of approximately 5–6 patients, and there is high variability in response rates across studies, ranging from 13–44%. The lack of standardized protocols for donor screening and stool preparation, unknown durability of therapeutic effects beyond 8–12 weeks in most studies, and safety concerns, including potential transmission of resistant organisms and long-term microbiome consequences, represent significant challenges.

Large-scale, multicenter, randomized controlled trials with standardized protocols are essential to validate the long-term safety, define optimal treatment regimens, and identify specific patient populations most likely to benefit from FMT. The therapy should currently be considered investigational for IBD outside of specialized centers with appropriate regulatory oversight and comprehensive monitoring capabilities. Current evidence suggests FMT may provide modest therapeutic benefit in carefully selected UC patients, particularly as adjunctive therapy in treatment-refractory cases, but it cannot yet be recommended as standard care given the limited magnitude of effect and critical need for protocol standardization [66,67]. Future research should focus on identifying predictive biomarkers, optimizing delivery methods, and developing next-generation engineered bacterial therapeutics that can provide more consistent and durable clinical benefits.

### 4.3. Gene Therapy and CRISPR-Cas9

Gene therapy is promising in treating inflammatory bowel disease (IBD) by targeting the underlying genetic and molecular pathways involved in disease pathogenesis. Complex interactions between genetic susceptibility, environmental factors, and immune dysregulation characterize IBD. Gene therapy aims to correct or modulate these pathways, offering the potential for a more targeted and sustainable therapeutic approach [73].

One of the primary strategies under investigation is the delivery of therapeutic genes that can suppress inflammatory pathways or promote mucosal healing. Gene editing technologies such as CRISPR/Cas9 have been explored for their ability to directly modify genes implicated in immune regulation, including those encoding cytokines or transcription factors involved in the inflammatory cascade [74]. Recent advances in base editing and prime editing have improved the precision and safety of gene editing approaches [75,76]. By downregulating pro-inflammatory cytokines such as TNF-α and IL-1β or enhancing the production of anti-inflammatory mediators like IL-10, gene therapy could theoretically achieve better control of inflammation in IBD patients, potentially reducing the reliance on immunosuppressive drugs [74].

In addition, gene therapies targeting epithelial barrier function are being explored. Gene transfer of tight junction proteins or mucosal repair molecules might restore the integrity of the intestinal epithelium, which is often compromised in IBD [77]. Recent studies have investigated the use of engineered bacteria as delivery vehicles for therapeutic genes, offering targeted delivery to the gut mucosa [78]. Preliminary studies in animal models and early-phase clinical trials have shown that gene therapy can potentially reduce mucosal damage and improve disease outcomes [77]. However, challenges remain, including the need for efficient and safe gene delivery systems and concerns over long-term and off-target effects.

Recent developments in lipid nanoparticle delivery systems and adeno-associated virus vectors have improved the safety and efficacy of gene delivery to intestinal tissues [79,80]. While gene therapy in IBD is still in its infancy, progress in gene editing tools and advances in delivery methods provide hope for the future of targeted and personalized treatments for IBD patients. Further research, particularly in larger clinical trials, is necessary to establish the feasibility, efficacy, and safety of gene therapy for IBD [73].

### 4.4. Stem Cell Therapies

Stem cell therapy is another innovative approach to treating inflammatory bowel disease (IBD) Table 3. It focuses on regenerative medicine to repair damaged gut tissue and modulate immune responses. The underlying premise is that stem cells, particularly mesenchymal stem cells (MSCs), can differentiate into various cell types, promote tissue repair, and modulate the immune system. This has led to significant interest in their potential to treat IBD, especially in patients with refractory disease or those not responding well to conventional therapies [81].

MSCs, which can be isolated from various sources such as bone marrow, adipose tissue, or umbilical cord blood, have been shown to possess anti-inflammatory properties. These cells can secrete various cytokines and growth factors that suppress excessive immune activation, promote tissue healing, and potentially restore gut mucosal integrity [82]. MSC-based therapies have demonstrated encouraging results in preclinical studies, with animal models showing improved healing of intestinal lesions, reduced inflammation, and restoration of intestinal barrier function [82].

In clinical settings, the ADMIRE-CD trial demonstrated significant efficacy of allogeneic adipose-derived MSCs for complex perianal fistulas in Crohn’s disease, leading to regulatory approval in Europe [83]. Small-scale trials have reported promising outcomes in luminal disease, with some studies showing improvements in disease activity and reduction in inflammatory markers [84]. Recent advances include the development of off-the-shelf allogeneic MSC products and combination approaches with scaffolds or growth factors [85,86].

However, the clinical results for luminal IBD have been mixed, and there is still uncertainty regarding the optimal type of stem cells to use, the method of administration (intravenous versus local injection), and the number of treatments required to achieve lasting benefits [87]. Recent studies have investigated induced pluripotent stem cells and organoid-based approaches for more personalized treatments [88].

Additionally, concerns remain about the long-term safety of stem cell therapies, particularly the risk of tumorigenicity or unintended differentiation of stem cells into inappropriate cell types. Although MSCs are generally considered safe, further investigation is needed to assess the risk of adverse events over time, especially in immunosuppressed IBD patients [89].

Recent regulatory guidelines have provided clearer pathways for stem cell therapy development, and ongoing Phase III trials will provide more definitive evidence of efficacy [90]. In conclusion, stem cell therapy represents a promising but evolving area in IBD treatment. With continued research, refinement of techniques, and larger, well-designed clinical trials, stem cell-based therapies may play an increasingly important role in managing IBD, particularly in patients with severe, refractory disease.

Emerging biologics modulate immune responses by targeting key cytokines: IL-12/23 (ustekinumab, lebrikizumab), IL-17A/F (secukinumab, ixekizumab), and IL-22 (fezakinumab). These cytokines affect T cell activation and gut inflammation.

## 5. Limitations and Future Directions

### 5.1. Current Limitations

Despite promising therapeutic advances, significant limitations persist in the evaluation and implementation of emerging IBD therapies. Current clinical trial designs often employ heterogeneous patient populations without adequate stratification for disease phenotype, genetic background, or microbiome composition, potentially masking therapeutic efficacy in specific patient subgroups [91]. Traditional endpoints focusing on clinical response may inadequately capture the complex, multidimensional nature of treatment success, particularly for novel interventions targeting tissue repair or microbiome restoration [92].

The lack of validated predictive biomarkers remains a critical limitation in personalizing emerging therapies. While promising candidates, including tissue transcriptomic signatures, serum metabolomics profiles, and microbiome diversity indices, have been identified, their clinical utility requires validation in large, prospective cohorts [93]. Long-term safety profiles for many emerging therapies remain incompletely characterized, particularly regarding malignancy risk, opportunistic infections, and effects on vaccination responses [94].

The high cost of innovative therapies presents a significant barrier to widespread implementation, particularly in resource-limited healthcare systems. The complexity of manufacturing and administration for advanced therapies may restrict their availability to specialized centers [95].

### 5.2. The Ceiling of Advanced Therapy: Understanding Therapeutic Plateaus

Despite significant advances in IBD management over the past two decades, a concerning pattern has emerged across all major therapeutic classes, creating what has been termed the “ceiling of advanced therapy.” This phenomenon represents the apparent maximum achievable clinical benefit with current therapeutic approaches, which consistently demonstrates similar efficacy profiles regardless of mechanism targeted [37,38,41,43]. Clinical trials reveal that most biologics achieve primary response rates in approximately two-thirds of patients, with clinical remission at one year maintained in roughly half of treated individuals, while primary non-response continues to affect a substantial proportion of patients and secondary loss of response occurs annually in a notable percentage regardless of the targeted pathway [38,41,43]. Recent network meta-analyses suggest remarkably similar efficacy profiles across mechanistically distinct therapies, with anti-TNF agents, anti-integrin therapy, anti-IL12/23 approaches, JAK inhibitors, and newer anti-IL23 agents demonstrating convergent clinical outcomes despite targeting different inflammatory pathways [37]. This convergence suggests that targeting individual inflammatory pathways may have reached its maximum therapeutic potential using current approaches.

Several factors likely contribute to this therapeutic ceiling. Disease heterogeneity encompasses multiple phenotypes with distinct genetic backgrounds, as genome-wide association studies have identified numerous risk loci associated with IBD pathways [19]. The complexity includes diverse immunologic signatures, varying microbiome compositions, and different environmental triggers, making single-target approaches insufficient to address this fundamental heterogeneity [17,18]. The immune system demonstrates remarkable adaptive capacity, potentially developing compensatory inflammatory pathways when single targets are blocked through cytokine redundancy, pathway switching, and tissue memory mechanisms that maintain inflammatory programs independent of systemic suppression [25]. Additionally, emerging evidence suggests non-immunologic disease drivers may contribute to disease perpetuation, including epithelial barrier dysfunction independent of immune activation, enteric nervous system alterations, and metabolic dysregulation affecting cellular energetics [28]. Current biologics also face inherent limitations including variable tissue penetration to affected mucosal sites, differences in drug clearance affecting therapeutic exposure, immunogenicity leading to neutralization, and population-based rather than individualized dosing regimens [38,41].

Breaking through this therapeutic ceiling likely requires precision medicine approaches utilizing biomarker-guided therapy selection, as evidenced by the clinical utility of pharmacogenomic testing and therapeutic drug monitoring [36,39,40]. Combination and sequential strategies may help overcome pathway redundancy through simultaneous pathway inhibition, sequential therapy optimization based on response patterns, and novel approaches targeting multiple mediators [62]. Future therapeutic development must likely move beyond traditional inflammatory targets to include tissue repair promotion, epithelial barrier restoration, metabolic pathway targeting, and neuromodulation approaches. Advanced delivery systems may overcome current pharmacokinetic limitations through targeted drug delivery, sustained-release formulations, cell-based therapies, and engineered biologics with improved tissue distribution.

### 5.3. Future Research Directions

Future clinical research should incorporate adaptive trial designs and master protocols evaluating multiple therapies within stratified patient populations [96]. The development of comprehensive diagnostic platforms combining multi-modal data analysis will enable precise patient stratification and treatment selection [97]. Machine learning algorithms incorporating genomic, transcriptomic, and microbiome data will facilitate predictive models for treatment response [98].

Systematic evaluation of combination therapies represents a critical research priority, with mathematical modeling helping identify synergistic combinations while minimizing toxicities [99]. Future microbiome research should focus on identifying specific bacterial strains and metabolites responsible for therapeutic effects, enabling standardized interventions [100].

## 6. Conclusions

The therapeutic landscape for inflammatory bowel disease has entered an unprecedented era of innovation, characterized by diverse mechanistic approaches that collectively address the complex, multifactorial nature of IBD pathogenesis. This comprehensive review highlights several key innovative paradigms that are reshaping our approach to IBD management.

### 6.1. Precision Medicine and Biomarker-Guided Therapy

The most transformative innovation lies in the shift toward precision medicine, where treatment selection is guided by individual patient characteristics including genetic profiles, immunologic signatures, and microbiome composition. Pharmacogenomic testing for thiopurine metabolism, anti-drug antibody monitoring for biologics, and emerging metabolomic biomarkers represent the foundation of personalized IBD care [36,39]. The integration of artificial intelligence algorithms for treatment response prediction promises to optimize therapeutic decision-making and minimize treatment failures [15,16].

### 6.2. Mechanistic Diversification Beyond TNF α

The expansion beyond anti-TNF therapy has yielded multiple successful therapeutic targets, each addressing distinct aspects of IBD pathogenesis. IL 23 inhibition with agents like mirikizumab has demonstrated superior efficacy in maintaining long-term remission, while JAK inhibition provides a rapid onset of action through intracellular pathway modulation [52,60]. S1P receptor modulators offer gut-selective immunomodulation with favorable safety profiles, representing a new class of oral therapeutics [55].

### 6.3. Microbiome as a Therapeutic Target

The recognition of gut microbiota as both a disease driver and therapeutic target has opened entirely new treatment avenues. Fecal microbiota transplantation has evolved from experimental therapy to evidence-based treatment for selected UC patients, while standardized frozen preparations have improved accessibility [71,72]. Next-generation engineered bacterial therapeutics represent the convergence of synthetic biology and IBD treatment.

### 6.4. Regenerative Medicine and Tissue Repair

Stem cell therapies have demonstrated clinical efficacy in complex perianal Crohn’s disease through the successful ADMIRE CD trial, leading to regulatory approval in Europe [83]. The integration of tissue engineering approaches with organoid technology represents the future of regenerative IBD therapy, potentially addressing the fundamental tissue damage that underlies disease chronicity [88].

### 6.5. Dual and Multi-Target Approaches

The concept of simultaneously targeting multiple inflammatory pathways represents a paradigm shift from single-agent therapy. Bispecific antibodies and combination biologics offer the potential for superior efficacy in treatment-refractory patients while potentially reducing the risk of resistance development [63].

### 6.6. Regulatory and Implementation Advances

Recent FDA approvals of mirikizumab and etrasimod demonstrate accelerated regulatory pathways for innovative IBD therapies [55,60]. The development of clearer regulatory guidelines for advanced therapies like FMT and stem cell treatments has facilitated clinical development [90].

The convergence of these innovative approaches suggests that the future of IBD management will be characterized by highly personalized, mechanism-based treatment algorithms that optimize efficacy while minimizing toxicity. The ultimate goal, achieving sustained remission with restoration of normal immune function and gut barrier integrity, appears increasingly achievable through these emerging therapeutic paradigms.

## Figures and Tables

**Table 1:** Overview of Current Biologic Therapies in IBD.

Therapy	Mechanism of Action	Indication	Efficacy (Induction)	Efficacy (Maintenance)	Safety Profile
Infliximab	Anti-TNFα monoclonal antibody	CD, UC	60–80%	40–60%	Infusion reactions, infections, and malignancy
Adalimumab	Anti-TNFα monoclonal antibody	CD, UC	60–70%	50–60%	Injection site reactions, infections, and malignancy
Vedolizumab	Anti-α4β7 integrin monoclonal antibody	CD, UC	50–60%	45–60%	Infusion reactions, infections, and rare PML risk
Ustekinumab	IL-12/IL-23 inhibitor	CD, UC	60–70%	55–70%	Headache, fatigue, upper respiratory infections

**Table 2:** Small-Molecule Therapies in IBD.

Therapy	Mechanism of Action	Indication	Efficacy	Safety Profile
Tofacitinib	Janus kinase (JAK) inhibitor	UC	Induction: 60–65% Maintenance: 50–55%	Risk of infections, malignancy, and thrombosis
Ozanimod	S1P receptor modulator	UC	Induction: 60% Maintenance: 45–50%	Increased liver enzymes, infections, and PML
Filgotinib	Janus kinase (JAK) inhibitor	CD	Induction: 50–60% Maintenance: 45–50%	Infections, liver toxicity
Etrasimod	S1P receptor modulator	UC	Induction; 55–60% Maintenance; 50–55%	Bradycardia, liver enzyme elevation

**Table 3:** Overview of Emerging Therapies in IBD.

	Target/Mechanism	Indication	Clinical Status/Key Findings
FMT	Restoring gut microbial balance	UC > CD	Multiple positive RCTs; standardized preparations available
Gene Therapy and CRISPR Cas9	Editing immune-related genes (NOD2, IL 10)	Refractory IBD	Preclinical development; improved delivery systems
Stem Cell Therapies	Immunomodulation tissue repair	Perianal CD, Refractory IBD	ADMIRE CD trial supports efficacy; regulatory approval in Europe
Anti-IL 17 Biologics	Inhibit IL-17	CD	Phase II trials; mixed efficacy; safety concerns
Anti-IL 23 Biologics	Inhibit IL-23 (p19 subunit)	UC & CD	Mirikizumab FDA approved 2023; Phase III trials ongoing
Dual Targeting Therapies	Anti-TNF + Anti-IL 23 agents	Severe/refractory IBD	Early phase trials; bispecific antibodies in development

## Data Availability

No data were reported.
